# Nucleotide receptor P2RY4 is required for head formation via induction and maintenance of head organizer in *Xenopus laevis*

**DOI:** 10.1111/dgd.12563

**Published:** 2018-08-01

**Authors:** Ayano Harata, Mika Hirakawa, Tetsushi Sakuma, Takashi Yamamoto, Chikara Hashimoto

**Affiliations:** ^1^ JT Biohistory Research Hall Takatsuki Japan; ^2^ Department of Mathematical and Life Sciences Graduate School of Science Hiroshima University Higashi‐Hiroshima Japan; ^3^ Department of Biological Sciences Graduate School of Science Osaka University Toyonaka Japan

**Keywords:** head formation, head organizer, P2Y receptor, *Xenopus laevis*

## Abstract

Vertebrates have unique head structures that are mainly composed of the central nervous system, the neural crest, and placode cells. These head structures are brought about initially by the neural induction between the organizer and the prospective neuroectoderm at early gastrula stage. Purinergic receptors are activated by nucleotides released from cells and influence intracellular signaling pathways, such as phospholipase C and adenylate cyclase signaling pathways. As P2Y receptor is vertebrate‐specific and involved in head formation, we expect that its emergence may be related to the acquisition of vertebrate head during evolution. Here, we focused on the role of *p2ry4* in early development in *Xenopus laevis* and found that *p2ry4* was required for the establishment of the head organizer during neural induction and contributed to head formation. We showed that *p2ry4* was expressed in the head organizer region and the prospective neuroectoderm at early gastrula stage, and was enriched in the head components. Disruption of *p2ry4* function resulted in the small head phenotype and the reduced expression of marker genes specific for neuroectoderm and neural border at an early neurula stage. Furthermore, we examined the effect of *p2ry4* disruption on the establishment of the head organizer and found that a reduction in the expression of head organizer genes, such as *dkk1* and *cerberus*, and *p2ry4* could also induce the ectopic expression of these marker genes. These results suggested that *p2ry4* plays a key role in head organizer formation. Our study demonstrated a novel role of *p2ry4* in early head development.

## Graphical abstract

P2Y4 receptor is required for the formation of head organizer and function in the following induction process of neural and neural crest. We demonstrated a novel role of p2ry4 in early head development.

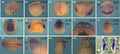

## 1 INTRODUCTION

Characteristic head structures are thought to define vertebrate species. The vertebrate head structure is basically derived from the brain, the neural crest, and placode cells. Vertebrate head formation originates from the establishment of an organizer (Niehrs, 2004; Spemann & Mangold, 1924, 2001). At the onset of gastrulation, the organizer is formed in the dorsal equatorial region. The organizer ectopically induces dorsal structures, including the central nervous system, when transplanted ventrally (Spemann & Mangold, 1924, 2001). The organizer can be subdivided into two regions: the head and trunk organizers (Sasai & De Robertis, 1997). BMP antagonists (Noggin, Chordin, and Follistatin) emanating from the organizer directly induce neural tissues in the ectoderm layer (Fainsod et al., 1997; Sasai et al., 1994; Smith & Harland, 1992). As the induced neural tissues are posteriorized by Wnt signal, Wnt antagonists, such as Dkk1 and Cerberus (Cer), are secreted from the head organizer to establish the head neural tissues (brain) (Glinka, Wu, Onichtchouk, Blumenstock, & Niehrs, 1997; Piccolo et al., 1999). When neural induction occurs in early gastrula, the neural crest and placode cells, which are necessary for constructing most of the head structures, are also induced at the boundary between neural plate and epidermal ectoderm (Nieto, 2001). It was proposed that the vertebrate head evolved from invertebrates through the emergence of cranial neural crest and placode (Gans & Northcutt, 1983). The vertebrate head is made from the derivatives of cranial neural crest and placode, meaning that the acquisition of these tissues and the emergence of vertebrate species may be correlated.

Purinergic P2Y receptors are G‐protein coupled receptors (GPCRs) and known to control various signal transduction pathways, including the activation of phospholipase C (PLC), the formation of inositol phosphate, the release of intracellular calcium, and the inhibition of cAMP synthesis (Abbracchio et al., 2006), by mediating responses to extracellular nucleotides (Abbracchio & Burnstock, 1994; Burnstock & Kennedy, 1985). In human, eight P2Y receptors, P2RY1, P2RY2, P2RY4, P2RY6, P2RY11, P2RY12, P2RY13, and P2RY14, are known (Abbracchio et al., 2003). In *X. laevis*, ten P2Y receptors have been identified: P2RY1, P2RY2, P2RY4, P2RY6, P2RY8, P2RY10, P2RY11, P2RY12, P2RY13, and P2RY14. Human P2Y receptors are divided into two subgroups on the basis of phylogenetic and structural characteristics (Abbracchio et al., 2003). Interestingly, P2Y receptors exist in only vertebrates and not invertebrates. Several vertebrate P2Y receptors form three separate groups (Bhatnagar, Mishra, & Pathak, 2011).

P2Y receptors have been investigated from both physiological and pathophysiological perspectives, but little is known about how they function in early development. Some P2Y receptors are known to function in processes related to head morphogenesis. For example, two *Xenopus* G‐protein coupled purinergic receptors, *p2ry1* and *p2ry11*, are expressed in the prospective head region and involved in head formation (Harata, Nishida, Nishihara, & Hashimoto, 2016), and *p2ry1* synergistically regulates eye development with the ectonucleoside triphosphate diphosphohydrolase (E‐NTDPase) (Masse, Bhamra, Eason, Dale, & Jones, 2007). Furthermore, lysophosphatidic acid receptor 6 (*lpar6*), also known as *p2ry5*, is required for forebrain development (Geach et al., 2014). In *X. laevis* tadpole, pyrimidinergic receptor P2Y, G‐protein coupled, 4 L homeolog, *p2ry4.L*, is expressed in basal cells of the main olfactory epithelium and involved in the regulation of cell turnover in the olfactory epithelium (Hassenklover, Schwartz, Schild, & Manzini, 2009; Hassenklover et al., 2008). A study provided evidence that the supporting cells of the vomeronasal organ express *p2ry4.L* and the basal cells express multiple P2Y receptors (Dittrich, Sansone, Hassenklover, & Manzini, 2014).

We show herein the involvement of P2RY4 receptor in head formation in *Xenopus* development. *p2ry4* is expressed in tissues necessary for head formation, such as the head organizer, the anterior neural tissues, the head neural crest, and placode cells, during *Xenopus* early development. Loss of *p2ry4* function impaired head structure development, revealing the functional involvement of *p2ry4* in head formation. *p2ry4* seems to function in multiple steps of head formation, such as involution movement, head organizer formation, and neural crest and placode cell establishment. In addition, P2Y receptors are highly conserved in vertebrates and their homologs cannot be found in any invertebrates. Taken together, as the head structure is thought to define the vertebrate (Ota, Kuraku, & Kuratani, 2007), it might not be a simple coincidence that the vertebrate‐specific receptor is involved in the formation of a vertebrate‐specific structure, namely, “the head”. This has further motivated us to think of how vertebrate species emerged in the course of evolution.

## 2 MATERIAL AND METHODS

### 2.1 Embryo manipulation

Adult *Xenopus laevis* was purchased from Watanabe Zosyoku (Hyogo, Japan) and embryos were obtained by artificial fertilization. Oocytes were obtained from female injected with 500 IU of human chorionic gonadotropin (ASKA Pharmaceutical) 15‐hr earlier, and fertilized with minced testis. The jelly coat was removed by treatment with 3% cysteine (pH 8.0) and the embryos were maintained in 10% Steinberg's solution (1× Steinberg's solution: 58 mM NaCl, 0.67 mM KCl, 0.34 mM Ca(NO_3_)_2_·4H_2_O, 0.83 mM MgSO_4_·7H_2_O, 10 mM HEPES, pH 7.3 at 23°C) until the described stages. The developmental stages were determined according to Nieuwkoop and Faber (Nieuwkoop & Faber, 1956).

### 2.2 Design and construction of TALEN vectors

Platinum TALEN plasmids were constructed using a Platinum Gate TALEN Kit (Addgene, Kit #1000000043), as previously described (Sakuma, Ochiai et al., 2013). ptCMV‐153/47‐VR vectors were used as destination vectors. The target sequences are as follows (uppercase and lowercase letters indicate target sequence and spacer region, respectively): *p2ry4.L*, 5′‐TCATGGCCACTTCCTACCctactttccttacaaCCCCCTACCTGCCGATGA‐3′. TALEN mRNAs were synthesized from TALEN constructs linearized by *Xma*Ι digestion using an mMESSAGE mMACHINE T7 Ultra Kit (AM1345, Ambion).

### 2.3 Mutation analysis

Genomic DNA extraction from embryos was carried out at 60°C for 3 hr using an extraction buffer (TE; 10 mM Tris‐HCl (pH 8.0), 1 mM EDTA (pH 8.0), 2.5% SDS, 0.25 mg/ml ProK) and this was followed by purification. Uninjected embryos were collected at gastrula (*n* = 10), neurula (*n* = 10), and tailbud (*n* = 10) stages. TALEN target site was amplified by specific primers using LA *Taq* DNA polymerase (RR002A, TAKARA). The PCR primers are as follows: 5′‐CCTCACAGCAAGCATACTGACAAAGC‐3′ and 5′‐ACGTACAATGTGTCCGACAGTGCAAGG‐3′. PCR products were purified by Sephacryl S‐300 (17‐0599‐01, GE Healthcare Life Sciences) chromatography and subcloned into pCS2AT‐T+ by TA cloning. pCS2AT‐T+ was constructed by inserting the sequence (5′‐CCAGATCTTTCCTGGCGGCCGCTTTCCATTACAAGCTTGG‐3′) into the EcoRV site of pCS2AT+ (Yamaguti, Cho, & Hashimoto, 2005). Positive clones were selected by colony PCR and the colony PCR products were sequenced using a BigDye Terminator V3.1 Sequencing Kit (BigDye).

### 2.4 DNA constructs and mRNA preparation

*Xenopus laevis* cDNA clones were obtained from Xenbase (http://www.xenbase.org/entry/) and cloned into the pCS2AT+ vector; NM_001086874 (*p2ry4.L*) and NM_001086456 (*snail1*). *hairy2b* (Tsuji, Cho, & Hashimoto, 2003), *dkk1*,* cerberus* (Yamaguti et al., 2005), *krox20* (Nieto, Bradley, & Wilkinson, 1991), *otx2* (Blitz & Cho, 1995), *pax6*,* rx1* (Murato & Hashimoto, 2009), and *sox2* (Nagatomo & Hashimoto, 2007) were previously described. *brachyury* was a gift from K. Cho; β*‐catenin*, from D. Tunner; *Chordin*, from Y. Sasai; *Noggin*, from R. Harland; and *TBRII*, from K. Umesono. Primers of pCS2AT+ plasmid containing *p2ry4.L* are shown below: *p2ry4.L*‐F (5′‐GGGATCGATCCACCATGACTGAGGACATCATGGC ‐3′) and *p2ry4.L*‐R (5′‐GGGCTCGAGTTATGCCTTTGGGAAGTTCTG ‐3′). Capped mRNAs were synthesized by an mMESSAGE mMACHINE sp6 Kit (AM1340, Ambion).

### 2.5 Whole‐mount in situ hybridization

Embryos were collected and fixed in MEMFA and whole‐mount in situ hybridization (WISH) was performed as previously described (Harland, 1991) with minor modifications. For the chromogenic reaction, nitroblue tetrazolium (NBT) (11383213001, Roche)/5‐bromo‐4‐chloro‐3‐indoxyl phosphate (BCIP) (11383221001, Roche) was used as the substrate of alkaline phosphatase. Antisense RNA probe templates were prepared from the above‐mentioned constructs. Digoxigenin‐labeled‐antisense RNAs were generated by in vitro transcription with a MAXIscript Kit (Ambion) and DIG RNA Labeling Mix (11277073910, Roche).

## 3 RESULTS

### 3.1 Spatial expression patterns of p2ry4

*Xenopus laevis* is known to be allotetraploid species, and the L and S chromosomes exist in *X. laevis* genome. However, genes have been lost asymmetrically between the two subgenomes. Some genes are lost from one subgenome and arose singleton, and the size and number of deletion are greater on the S subgenome (Session et al., 2016). We confirmed that *p2ry4* exist only in L chromosome and no other *p2ry4* related sequence could be found in S chromosome. Therefore, in this report we call *p2ry4.L* just *p2ry4*. First, we performed a phylogenetic analysis of P2Y receptors. P2Y receptors were found in only vertebrate and some ancestral genes were found in lamprey (see Discussion, Supporting information Figure S1). Some P2Y receptors were involved in the head morphogenesis of *X. laevis* (for example, see Masse et al., 2007; Hassenklover et al., 2008; Geach et al., 2014). We also reported that *p2ry1* and *p2ry11* were involved in the head formation (Harata et al., 2016). Furthermore, the phylogenetic analysis showed that these genes were classified into four groups and *p2ry1*,* p2ry2*,* p2ry4*,* p2ry6* and *p2ry11* were included in the same group.

**Figure 1:**
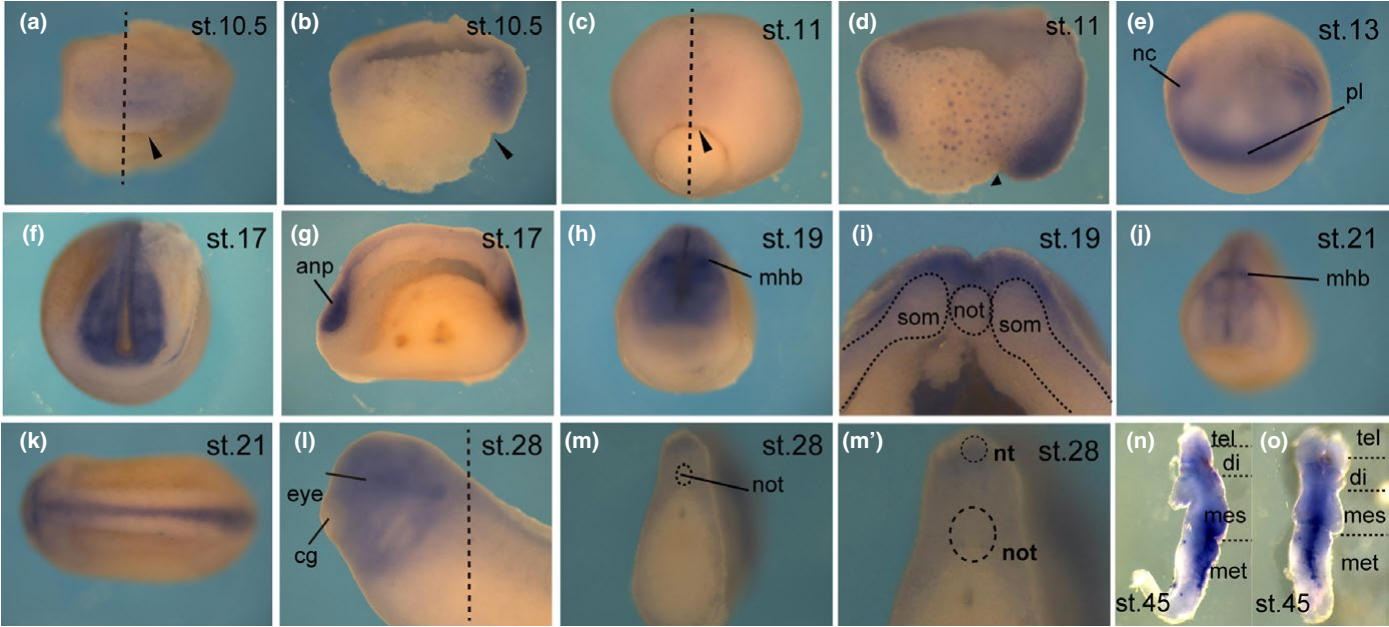
Expression patterns of *p2ry4* during early development. WISH of *p2ry4* from gastrula to tailbud stage. Stages 10.5 and 11 (a and c, dorsal views; b and d, sagittal hemisections). *p2ry4* was expressed in the prospective neuroectoderm and the involuting dorsal and ventral mesoderm at gastrula stage. At stage 10.5, *p2ry4* was prominently expressed in the head organizer region (b). Stages 13, 17, 19, and 21 (e, f, h, j, anterior views; k, dorsal view; g, sagittal hemisection; i, transverse section). Stage 28 (l, lateral view; m, transverse section; m’, high‐magnification image of m). The extirpated brain of tadpole (n, lateral side; o, dorsal side). nc, neural crest; pl, placode cell; anp, anterior neural plate; mhb, midbrain‐hindbrain boundary; nt, neural tube; not, notochord; som, somite; cg, cement gland; tel, telencephalon; di, diencephalon; mes, mesencephalon; met, metencephalon. Dotted straight line indicates section plane. Black arrowheads indicate blastopore

Then, to clarify whether *p2ry4*, whose ancestral gene was found in lamprey, is actually related to the *Xenopus* head formation, we performed WISH. *p2ry4* was weakly expressed in the dorsal marginal zone at blastula stage (data not shown) and clearly detected in the dorsal and ventral marginal zone from early gastrula stage, the strongest expression being above the dorsal blastopore lip (Figure 1a, c). Bisected embryos revealed *p2ry4* expression in the dorsal and ventral involuting mesoderm and the prospective neuroectoderm (Figure 1b, d). At stage 10.5, *p2ry4* expression was observed in the inner tissue adjacent to the ectoderm via Brachet's cleft in the dorsal equatorial region (Figure 1b). The tissue is known to function as the head organizer (Yanagi et al., 2015). At early neurula stage, *p2ry4* was localized to the neural plate border, including the neural crest and placode cells, and was weakly expressed in the entire neural plate (Figure 1e). *p2ry4* gradually condensed into the anterior neural fold (Figure 1f) and was detected in the anterior neural plate (Figure 1g) at mid‐neurula stage. After the neural closure, *p2ry4* was distinctly detected in the prospective midbrain–hindbrain boundary (Figure 1h) and the neural groove (Figure 1i). At later stages, *p2ry4* transcripts were restricted in the central nervous system (Figure 1j–m). It was previously reported that *p2ry4* was specifically expressed in neural crest cells from early to mid‐neurula stage and in the discrete domain of the central nervous system at later stages (Bae et al., 2014). At tailbud stage, *p2ry4* was weakly expressed in the anterior neural tissues and the neural tube (Figure 1l, m) but was not detected in the notochord (Figure 1m, m’). Afterward, *p2ry4* was observed in the brain, specifically the mesencephalon and the metencephalon (Figure 1n, o). Thus, *p2ry4* was abundantly expressed in regions important for head formation during early development.

### 3.2 p2ry4 disruption in TALEN mRNA injected embryos

As shown above, *p2ry4* is expressed in regions important for head morphogenesis. To investigate the role of *p2ry4* in early development, first, we used a knockdown approach by using morpholino antisense oligonucleotides (MOs). However, we could not downregulate *p2ry4* function sufficiently due to the presence of splicing variants (Supporting information Figure S2), and thus we performed the targeted gene disruption of *p2ry4* by transcription activator‐like effector nuclease (TALEN) (Sakane et al., 2014; Sakuma & Woltjen, 2014; Sakuma, Hosoi et al., 2013; Suzuki et al., 2013). We injected 50 pg each of left and right TALEN pair (TALEN‐L/R) into fertilized eggs at 1‐cell stage, and 100 pg of left (TALEN‐L) or right (TALEN‐R) as negative control. Approximately 50% of TALEN‐L/R injected embryos died during gastrulation, whereas the rest of the injected embryos survived to the early tailbud stage (Supporting information Figure S3A). On the other hand, embryos treated with TALEN‐L (Supporting information Figure S3A) or TALEN‐R alone (data not shown) developed normally and their survival rates were consistently higher than 90%, indicating that TALEN mRNAs did not exhibit toxicity under our experimental conditions. Phenotypes of TALEN‐L/R injected embryos were divided into two groups: moderate and severe. The moderate group showed the small head phenotype (Supporting information Figure S3A, L/R‐1) and the severe group exhibited a defect in blastopore closure that led to the spina bifida phenotype with a small head (Supporting information Figure S3A, L/R‐2). Then, we determined whether the target gene was disrupted or not by DNA sequencing analysis (Supporting information Figure S3B) and found that *p2ry4* TALEN possessed the ability to disrupt the target gene at an early stage. Therefore, our results suggested that *p2ry4* plays a crucial role in early development.

### 3.3 p2ry4 is required for neural crest and placode development

**Figure 2:**
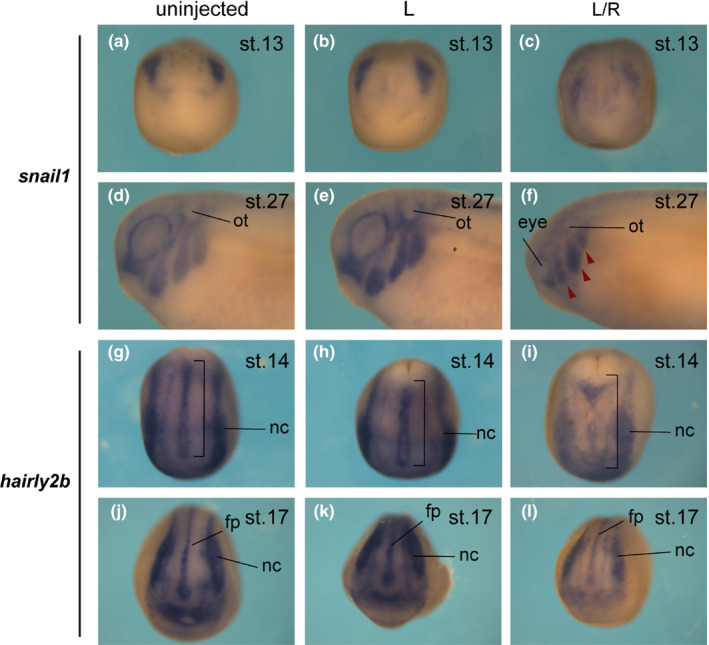
Loss of *p2ry4* affected neural crest and placode development. Uninjected (a, d, g, j), TALEN‐L injected (b, e, h, k), and TALEN‐L/R injected (c, f, i, l) embryos were subjected to WISH for *snail1* (a–f) and *hairy2b* (g–l). *Snail1* expression at early neurula (a–c) and early tailbud (d–f) stages. *Snail1* expression was reduced in TALEN‐L/R injected embryos (c, f, red arrowheads). *hairy2b* expression at early (g–i) and middle (j–l) neurula stages. *hairy2b* expression at the anterior neural plate border was reduced in TALEN‐L/R injected embryos. Anterior (a–c, j–l), lateral (d–f), and dorsal (g–i) views. ot, otic vesicle; nc, neural crest; fp, floor plate. Red arrowheads indicate migrating neural crest cells. Brackets indicate prospective floor plate

*p2ry4* was prominently detected in the neural crest (Figure 1e, f) and the knockout phenotype was exhibited particularly in the head region. Thus, first, we examined the influence of *p2ry4* depletion on the expression of neural crest marker *snail1* at neurula and tailbud stages (Figure 2a–f). *Snail1* expression was dramatically reduced in TALEN‐L/R injected embryos at early (100%, *n* = 12, Figure 2c) and mid‐neurula stages (data not shown), and the reduction of *snail1* expression was also observed in the branchial arch at tailbud stage (100%, *n* = 10, Figure 2f, arrowheads), indicating that the reduction of *snail1* expression in neural crest at neurula stage resulted in the loss of neural crest derivatives. The disruption of *p2ry4* also brought about such head defects as small eyes and incomplete formation of otic vesicles at early tailbud stage (Figure 2f). Furthermore, *p2ry4* was localized to the neural plate border, including the neural crest and placode cells at early neurula stage (Figure 1e), so we examined the expression of neural plate border marker *hairy2b* (Tsuji et al., 2003) in TALEN‐L/R injected embryos (Figure 2g–l). *hairy2b* is expressed in head and trunk neural crest (Nagatomo & Hashimoto, 2007; Tsuji et al., 2003). The loss of *p2ry4* reduced hairy2b expression in the anterior portion of the neural plate border at neurula stage in TALEN‐L/R injected embryos (71.4%, *n* = 14, Figure 2i; 81.8%, *n* = 11, Figure 2l), and *snail1* expression was also reduced in head neural crest. Taken together, the results suggested that *p2ry4* is important for the establishment of head neural crest and placode cells. The expression of *snail1* (*n* = 7, Figure 2a; *n* = 14, Figure 2b; *n* = 6, Figure 2d; *n* = 6, Figure 2e) or *hairy2b* (*n* = 8, Figure 2g; *n* = 11, Figure 2h; *n* = 6, Figure 2j; *n* = 9, Figure 2k) in TALEN‐L injected embryos showed the same patterns as that in uninjected embryos at neurula and tailbud stages, confirming that TALEN mRNA did not affect the development. These data indicated that *p2ry4* perturbation resulted in the defect of main head components, such as the neural crest and placode cells, yielding the small head phenotype.

### 3.4 p2ry4 disruption brings about defect in neural development

**Figure 3:**
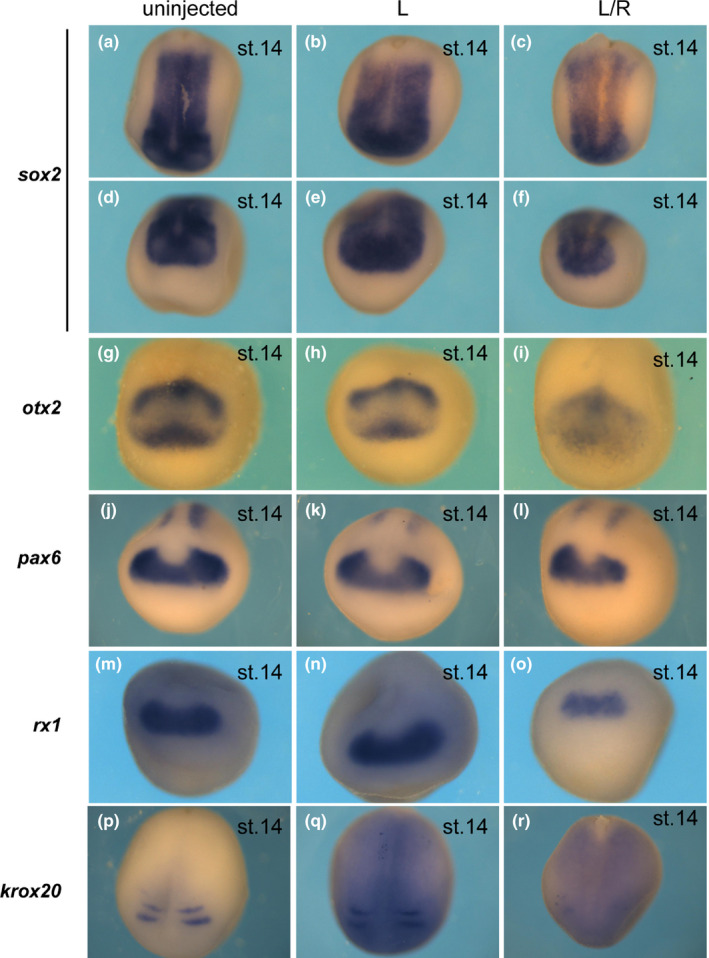
Loss of *p2ry4* affected early neural development. Uninjected (a, d, g, j, m, p), TALEN‐L injected (b, e, h, k, n, q), and TALEN‐L/R injected (c, f, i, l, o, r) embryos were subjected to WISH for *sox2* (a–f), *otx2* (g–i), *pax6* (j–l), *rx1* (m–o), and *krox20* (p–r) at early neurula stage. The expression of all the neural marker genes was reduced in TALEN‐L/R injected embryos. Dorsal (a–c, p–r) and anterior (d–o) views

To further examine the effect of *p2ry4* depletion on head development, we collected embryos at early neurula stage and analyzed the expression of several neural marker genes (Figure 3). We found that TALEN‐L/R injected embryos expressed neural plate marker *sox2*:* sox2* was markedly reduced in the anterior region (84%, *n* = 25, Figure 3c, f) compared to uninjected (*n* = 12, Figure 3a, d) or TALEN‐L injected (*n* = 17, Figure 3b, e) embryos. We also observed significant reductions in the expression of fore‐ to midbrain marker *otx2* (100%, *n* = 15, Figure 3i), retinal marker *pax6* or *rx1* (*pax6*, 83.3%, *n* = 12, Figure 3l; *rx1*, 90.1%, *n* = 22, Figure 3o), and hindbrain marker *krox20* (100%, *n* = 8, Figure 3r) in TALEN‐L/R‐injected embryos, whereas TALEN‐L injection had no effect on the expression of neural marker genes (*otx2*,* n* = 8, Figure 3g, *n* = 4, Figure 3h; *pax6*,* n* = 7, Figure 3j, *n* = 8, Figure 3k; *rx1*,* n* = 3, Figure 3m, *n* = 8, Figure 3n; *krox20*,* n* = 5, Figure 3p, *n* = 5, Figure 3q). These data indicated that the disruption of *p2ry4* affected anterior neural tissue development and the defects of neural tissue development were consistent with the small head phenotype. Thus, the reduction of neural marker genes at early neurula stage raised the possibility that *p2ry4* depletion might lead to the inhibition of neural induction during gastrulation.

### 3.5 p2ry4 is important for head organizer formation

**Figure 4:**
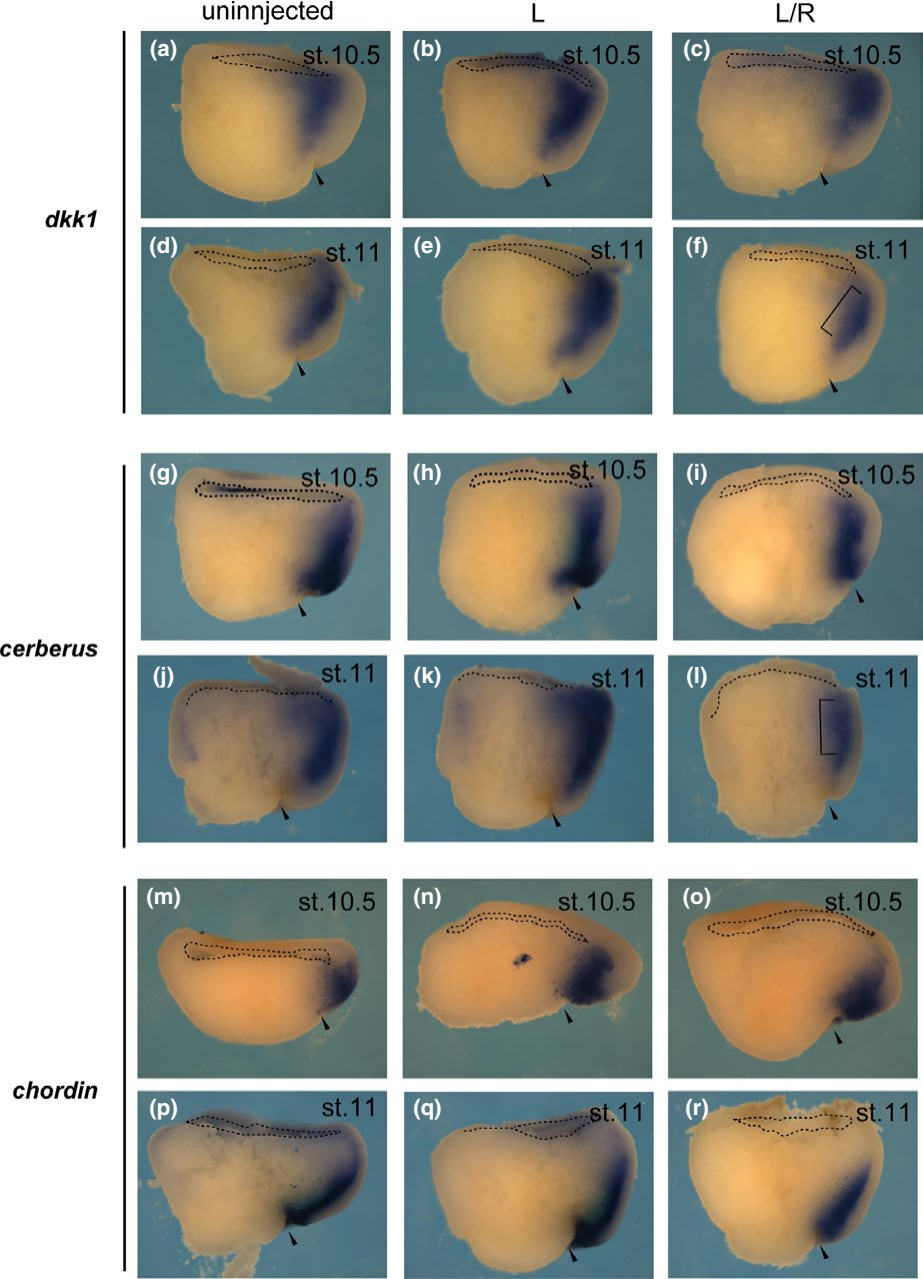
Disruption of *p2ry4* reduced expression of head organizer genes. Uninjected (a, d, g, j, m, p), TALEN‐L injected (b, e, h, k, n, q), and TALEN‐L/R injected (c, f, i, l, o, r) embryos were subjected to WISH for *dkk1* (a–f), *cerberus* (g–l), and *chordin* (m–r) at gastrula stage. The expression of *dkk1* and *cerberus* was reduced in TALEN‐L/R injected embryos at mid‐gastrula stage (f, l, brackets). The change of *chordin* expression in TALEN‐L/R injected embryos was not significant (o, r) compared to that in uninjected and TALEN‐L injected embryos (m, n, p, q). Dorsal halves of sagittally fractured embryos are shown. Dashed lines indicate the edge of blastocoel. Brackets indicate the area that were reduced in the expression of *dkk1* and *cerberus*. Arrowheads indicate blastopore

**Figure 5:**
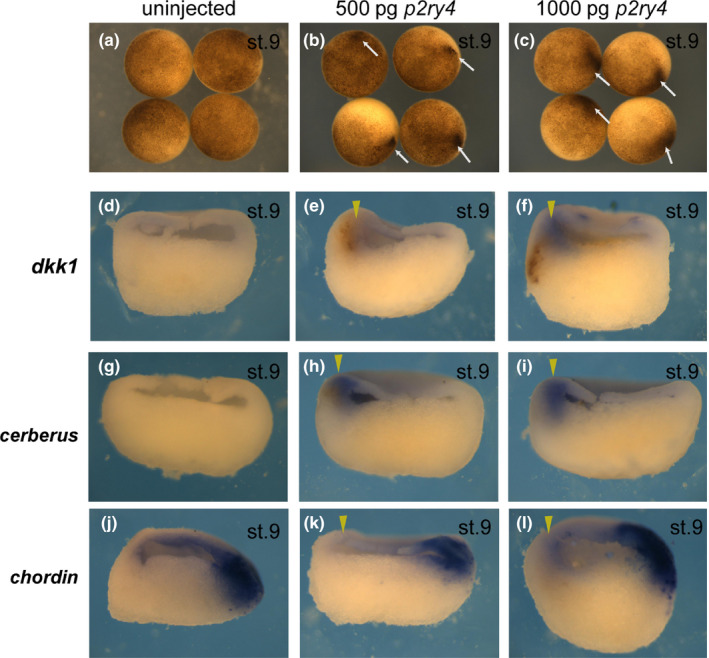
*p2ry4* resulted in ectopic expression of head organizer genes. No injection (a, d, g, j) and injection of 500 pg of *p2ry4* mRNA (b, e, h, k) and 1,000 pg of *p2ry4* mRNA (c, f, i, l) into the ventral side at 8‐cell stage and collection at blastula stage (a–c). Embryos were hemisectioned and subjected to WISH for *dkk1* (d–f), *cerberus* (g–i), and *chordin* (j–l). Overexpression of *p2ry4* led to the accumulation of pigment (b, c, arrows). *Dkk1* or *cerberus* expression was induced by *p2ry4* mRNA (e, f, h, i, arrowheads). Animal views (a–c). Arrowheads indicate injected sides

*p2ry4* was prominently observed in the head organizer region at early gastrula stage (Figure 1b). In order to clarify whether *p2ry4* functioned in the head organizer region during neural induction, we examined the effect of *p2ry4* depletion on the expression of organizer genes at gastrula stage (Figure 4). It is known that to determine epidermal fate in the presumptive ectoderm, BMP activities should be inhibited by such antagonists as Chordin, Noggin, and Follistatin, all of which are secreted by the organizer (Fainsod et al., 1997; Sasai et al., 1994; Smith & Harland, 1992). Then, head neural tissue is determined by the expression of such Wnt antagonists as *Dkk1* and *Cerberus* (*cer*) (Glinka et al., 1997, 1998; Piccolo et al., 1999). Although the expression of *dkk1* or *cer* in TALEN‐L/R injected embryos was hardly affected at early gastrula stage (*dkk1*, 11.8%, *n* = 17, Figure 4c; *cer*, 18.8%, *n* = 16, Figure 4i) (*dkk1*,* n* = 19, Figure 4a, *n* = 18, Figure 4b; *cer*,* n* = 8, Figure 4g, *n* = 9, Figure 4h), the reduction of expression of each marker gene in the TALEN‐L/R injected embryos was detected at mid‐gastrula stage (*dkk1*, 71.4%, *n* = 21, Figure 4f; *cer*, 89.5%, *n* = 19, Figure 4l) (*dkk1*,* n* = 17, Figure 4d, *n* = 15, Figure 4e; *cer*,* n* = 15, Figure 4j, *n* = 14, Figure 4k). This may suggest that *p2ry4* is necessary not for the induction but for the maintenance of *dkk1* and *cer* expression. On the other hand, we could not find any significant difference in *chordin* and *noggin* (data not shown) expression between TALEN‐L/R injected (*chordin*,* n* = 29, Figure 4o, *n* = 18, Figure 4r) and control (*chordin*,* n* = 25, Figure 4m, *n* = 23, Figure 4n, *n* = 9, Figure 4p, *n* = 14, Figure 4q) embryos. These data suggested that *p2ry4* may be specifically involved in the regulation of the expression of Wnt antagonists among organizer genes in the head organizer region. If this is true, *p2ry4* may be able to induce the expression of *dkk1* or *cer*. To examine this possibility, we injected 500 pg or 1,000 pg of *p2ry4* mRNA into one ventral animal cell at 8‐cell stage (Figure 5). The ectopic expression of *p2ry4* resulted in the pigment accumulation (Figure 5b, c, arrows) compared with uninjected embryos (Figure 5a) and thus, we were able to confirm that injected *p2ry4* was located in the area. We found that the expression of *dkk1* and *cer* was induced ectopically by *p2ry4* (*dkk1*,* n* = 15, Figure 5e, *n* = 16, Figure 5f; *cer*,* n* = 10, Figure 5h, *n* = 8, Figure 5i, arrowheads), whereas *chordin* expression was not induced (*n* = 7, Figure 5k, *n* = 7, Figure 5l, arrowheads). As endogenous *dkk1* and *cer* were not expressed in the dorsal side at blastula stage (*dkk1*, Figure 5d; *cer*, Figure 5g), we considered that *p2ry4* could induce the expression of these head organizer genes, possibly in a cell‐autonomous manner. These data suggested that *p2ry4* is required for the expression of Wnt antagonists, such as *dkk1* and *cer*, and may play a key role in the head organizer formation. Therefore, it is expected that the co‐injection of *p2ry4* with the truncated BMP receptor (*TBRII*) (Suzuki et al., 1994) would induce a complete secondary axis with head structures (Glinka et al., 1997) but not a secondary head structure (see Discussion and Supporting information Figure S4). Furthermore, the depletion of *p2ry4* resulted in the spina bifida phenotype (Supporting information Figure S3A, TALEN‐L/R‐2), which was caused by the defect of involution movement during gastrulation (Chung et al., 2004; Tao et al., 2005). Therefore, we analyzed the effect of *p2ry4* depletion on mesoderm involution by observing the expression patterns of *brachyury* (*bra*) as a chordamesoderm marker (Supporting information Figure S5) and *goosecoid* (*gsc*) as a prechordal mesoderm marker (Supporting information Figure S6) at gastrula stage. We found that *p2ry4* disruption inhibited the involution movement although mesoderm induction occurred (see Discussion and Supporting information Figures S5 and S6).

## 4 DISCUSSION

### 4.1 p2ry4 knockout using TALENs enables targeted mutagenesis in vivo for functional analysis

Our results revealed that the TALEN‐mediated knockout of *p2ry4* affected the expression of neural genes, head organizer genes, and the involution movement at gastrulation. Fifty percent of TALEN‐L/R injected embryos were lethal during gastrulation, whereas TALEN‐L or TALEN‐R injected embryos developed normally (Supporting information Figure S3A) and did not exhibit the mutation within the TALEN target site (Supporting information Figure S3B), indicating that TALEN mRNA itself did not show toxicity. Further, as we presented in Supporting information Figure S3, TALEN‐L/R injected embryos exhibited distinct developmental defects, such as high lethality, during the early developmental stage. We speculate that *p2ry4* is essential for the early development and the partial loss of *p2ry4* could induce a severe defect phenotype. To investigate the role of *p2ry4* in early development, first, we tried to use MO in the *p2ry4* knockdown experiments. RT‐PCR analysis indicated at least four splicing variants around the initiation codon (Supporting information Figure S2), suggesting difficulty of the functional inhibition of *p2yr4.L* by MO. In fact, the injection of MO designed for any one of the splicing variants could induce a moderate phenotype. However, the phenotypes of the MO‐injected embryos were basically the same as those of the TALEN‐L/R injected embryos, so we concluded that the phenotypes of the TALEN injected embryos could be a result of *p2ry4* depletion specifically. Interestingly, when all four MOs were injected at once, most of the embryos died during gastrulation. Although the blastopore appeared at the early gastrula stage in the four MO injected embryos and the TALEN injected embryos, normal blastopore closure was gradually suppressed. Therefore, it is possible that the complete loss of *p2ry4* may lead to developmental defect prior to gastrulation, as seen in the TALEN injected embryos.

### 4.2 p2ry4 is required for induction of neural tissues

We showed that *p2ry4* depletion resulted in the reduced expression of genes specific for the neuroectoderm (*sox2*,* otx2*,* pax6*,* rx1*, and *krox20*), the anterior neural border (*snail1* and *hairy2b*), and the head organizer (*dkk1* and *cer*). In addition, the ectopic expression of *p2ry4* induced the expression of the head organizer genes. These findings indicate that *p2ry4* primarily induces and/or maintains the expression of genes specific for the head organizer, and induces the formation of head neuroectoderm. Therefore, the incomplete establishment of the head organizer in *p2ry4* TALEN‐L/R injected embryos may result in the defect of anterior neural tissues. If this is the case, *p2ry4* should induce a complete secondary axis when expressed ventrally with BMP antagonists. To check this possibility, a truncated BMP receptor (*TBRII*) was injected with *cer* or *p2ry4* mRNA ventrally at the 8‐cell stage (Supporting information Figure S4). It is known that the inhibition of BMP by *TBRII* expression induces an ectopic dorsal structure (Suzuki et al., 1994) and the simultaneous inhibition of both BMP and Wnt signaling induces a complete secondary axis with head structures, including eyes and cement glands (Glinka et al., 1997). *TBRII* injection with *cer* induces a complete secondary axis (Piccolo et al., 1999). However, contrary to our expectation, *p2ry4* could not induce ectopic head structures when BMP signaling was suppressed (Supporting information Figure S4). These results suggested that *p2ry4* may be necessary but insufficient for the head organizer formation although *p2ry4* is required for the expression of *dkk1* and *cer*.

It was recently shown that two structurally and functionally related *Xenopus* GPCRs *flop1* (also known as *Xgpr4*; Chung et al., 2004) and *flop2* (also known as *xflop*; (Tao et al., 2005) induced the expression of Wnt antagonists, such as *dkk1* and *cer*, cell‐autonomously and functioned in head formation via an RhoA‐dependent or independent pathway (Miyagi, Negishi, Yamamoto, & Ueno, 2015). P2RY4 receptor is responsive to uridine nucleotide (UDP, UTP, and UMP) and ATP (Bogdanov, Dale, King, Whittock, & Burnstock, 1997), but *f1op1/2* ligands have not been identified yet. We expect that *p2ry4* would also have similar functions to *flop1/2* and contribute to head formation. Another *Xenopus* GPCR, *lpar6* (also known as *p2y5*; Choi et al., 2010), is required for telencephalon development (Geach et al., 2014). Thus, *p2ry4* co‐expressed with these genes may induce a complete secondary axis when *TBRII* is also expressed, whereas *p2ry4* alone cannot. Therefore, the head induction in *Xenopus* may be regulated by the synergistic effects of several GPCRs. Furthermore, even though the expression of *dkk1* and *cer* was not affected in TALEN‐L/R injected embryos at early gastrula stage, ectopic *p2ry4* expression induced the expression of these head organizer genes. We interpreted these contradictory observations as indicating that *siamois* and *twin*, the downstream targets of the early Wnt pathway, transiently induced *dkk1* and *cer* expression at early gastrula stage so that *p2ry4* depletion might not lead to the downregulation of *dkk1* and *cer*. The reduction of head organizer gene expression at mid gastrula stage in TALEN‐L/R injected embryos was also consistent with the possibility that *p2ry4* has the ability to maintain the expression of head organizer genes.

### 4.3 How does P2RY4 receptor function in head organizer formation?

In *X. laevis*, calcium signaling is suggested to be also involved in neural induction (Moreau, Neant, Webb, Miller, & Leclerc, 2008), in addition to BMP antagonists and fibroblast growth factors (Delaune, Lemaire, & Kodjabachian, 2005; Stern, 2005). P2Y receptors activated by nucleotides increase intracellular Ca^2+^ ([Ca^2+^]i) (Bogdanov et al., 1997), and in *X. laevis* tadpole, the extracellular nucleotides activate P2RY4 receptor in the supporting cells of the olfactory epithelium to increase [Ca^2+^]i (Dittrich et al., 2014; Hassenklover et al., 2008). Therefore, *p2ry4* observed in the prospective neuroectoderm or the head organizer region possibly causes an increase in [Ca^2+^]i during neural induction.

Ca^2+^ imaging in *Xenopus* embryos revealed a transient pattern of increase in [Ca^2+^]i in the dorsal ectoderm, and these [Ca^2+^]i increases occurred via the dihydropyridine (DHP)‐sensitive Ca^2+^ channel (DSCC) and were required for the conversion from ectoderm into neural fate (Moreau, Leclerc, Gualandris‐Parisot, & Duprat, 1994; Moreau et al., 2008). The involvement of [Ca^2+^]i increase in neural induction might be related not only to the dorsal ectoderm but also to the head organizer region, because DSCC was also expressed in the dorsal marginal zone at gastrula stage, and further misexpression of DSCC on the ventral side resulted in the ectopic expression of dorsal mesoderm genes, such as *chordin* and *cer*, and the dorsalized phenotype with a double axis (Palma, Kukuljan, & Mayor, 2001). Therefore, we speculate that the activated P2RY4 receptor may function to induce the expression of head organizer genes via the increase of [Ca^2+^]i. Furthermore, *xprmt1b*, a *Xenopus* homolog of mammalian arginine methyltransferase *prmt1*, was responsive to Ca^2+^ increase and expressed in prospective neural territories (Batut et al., 2005; Neant et al., 2015). *Xprmt1b* expression mediating DSCC affected the expression of neural precursor marker *zic3*. Thus, the elevation of [Ca^2+^]i via DSCC was suggested to participate in the neural induction and accordingly, calcium signaling through P2RY4 receptor might explain our finding that *p2ry4* was required for neural induction in the organizer region or the prospective neuroectoderm.

### 4.4 p2ry4 is involved in involution movement during Xenopus gastrulation

We also found that *p2ry4* depletion yielded the spina bifida phenotype, which was caused by a defect of tissue movement during gastrulation. *p2ry4* was expressed in the dorsal marginal zone, which was composed of the anterior and posterior mesoderm, suggesting that *p2ry4* might be also involved in such morphological movements as mesoderm involution and convergent extension. To examine the effect of *p2ry4* on involution movement at gastrula stage, we checked the expression patterns of *bra* (Supporting information Figure S5) and *gsc* (Supporting information Figure S6) and found that *p2ry4* disruption inhibited the involution movement without affecting the mesoderm induction. In normal development, the *gsc* domain internalized fully and the *bra* domain localized at the tip of the blastopore lip in mid gastrula embryos (Supporting information Figure S6D, E). However, in *p2ry4* depleted embryos, the *gsc* domain failed to internalize (Supporting information Figure S6F) and the anterior mesoderm faced the outside of embryo (Supporting information Figure S6I), suggesting that *p2ry4* was involved in the involution. It was reported that a member of the same cluster, *p2ry11*, was required for the convergent extension movement of the chordamesoderm, but not for the involution movement (Shindo et al., 2010). Therefore, *p2ry4* may participate in the mesoderm involution and then *p2ry11* cooperatively functions in the convergent extension.

### 4.5 p2ry4 may function in multiple steps of *X. laevis* head formation process

As mentioned above, we demonstrated that *p2ry4* functioned in neural induction through the appropriate formation of the head organizer. Embryos lacking the *p2ry4* function could not form the head structures properly. This means that the proper formation of the neural crest and placode cells is inhibited by the depletion of *p2ry4*. As neural crest formation is fully dependent on neural induction, it is quite natural to think that failure of head organizer formation can affect neural crest formation. In addition, *p2ry4* was prominently expressed at the neural boundary at neurula stage, and it was shown that *pax3* and *zic1* activated *p2ry4* as the target gene in neural crest cells (Bae et al., 2014), suggesting that this receptor might play a role in the establishment of neural crest identity. [Ca^2+^]i increases in response to ATP stimulation in various cell types were shown to play roles in regulating cell proliferation, differentiation, and migration (Berridge, 1993; Lauder, 1993). In *X. laevis*, multiple P2Y receptors were expressed in basal cells of the olfactory epithelium in which stem cells and various progenitors were present, and regenerative capacities were maintained. The increases of [Ca^2+^]i in basal cells were involved in the stimulation of cell proliferation to regenerate damaged tissues (Hassenklover et al., 2008, 2009; Hegg, Greenwood, Huang, Han, & Lucero, 2003). Neural crest cells are multipotent progenitor cells and need to be maintained in proliferative and undifferentiated states (Nagatomo & Hashimoto, 2007). In this regard, *p2ry4* condensed into the neural crest at neurula stage might regulate cell proliferation and differentiation to maintain progenitor cells.

### 4.6 Evolutionarily conserved p2y genes may be involved in acquisition of head in vertebrates

We found that P2Y receptors were highly conserved in vertebrates although their homologs were not present in any invertebrates, even in ascidian or amphioxus (Supporting information Figure S1). We found five ancestral p2y genes in lamprey and many paralogs in jawed vertebrate species. Phylogenetic analysis showed that p2y genes were classified into four clusters by comparative analysis of amino acid sequences. These four clusters might be generated by two rounds of whole genome duplication.

*p2ry1*,* p2ry4*, and *p2ry11* are classified in the same cluster (Supporting information Figure S1A). It has been shown that *p2ry1*,* p2ry4*, and *p2ry11* are involved in head formation (Harata et al., 2016; this report). In addition, *p2ry5*, which belongs to another cluster (Supporting information Figure S1D), is required for the initial specification and/or maintenance of the telencephalon (Geach et al., 2014). The expression patterns of *p2ry12*,* p2ry13*, and *p2ry14* are very similar to those of *p2ry1*,* p2ry4*, and *p2ry11* in various regions important for head formation (data not shown), suggesting that *p2ry12*,* p2ry13*, and *p2ry14* may be also required for head formation. Thus, the ancestral genes of these *p2y* could originally have crucial roles in head formation. It is known that vertebrates came to possess “the head” by acquiring the neural crest and placode cells during evolution (Gans & Northcutt, 1983). In addition, *p2y*, which is involved in head formation processes, is found only in vertebrates. This coincidence indicates that further studies of the role of P2Y receptors in head formation would give exciting hints to understand how vertebrate species emerged during evolution.
