# Supplementary figures and images for: Nucleotide receptor P2RY4 is required for head formation via induction and maintenance of head organizer in Xenopus laevis
Source: Dev Growth Differ. 2018 Aug 1;61(2):186–97. doi: 10.1111/dgd.12563 (PMC7379700; doi:10.1111/dgd.12563)

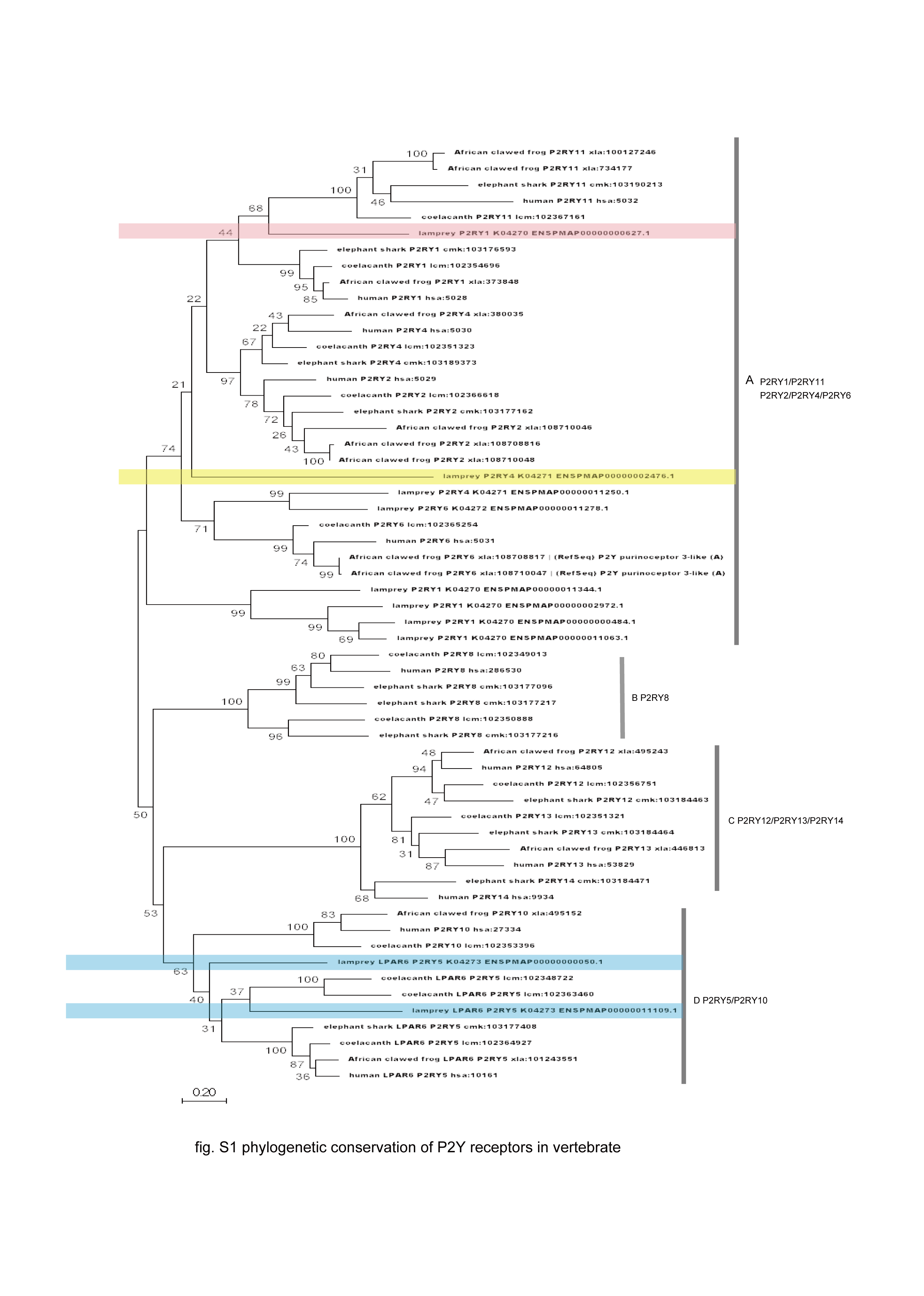

Supplement: Supplementary file 1 [file DGD-61-186-s001.tif]

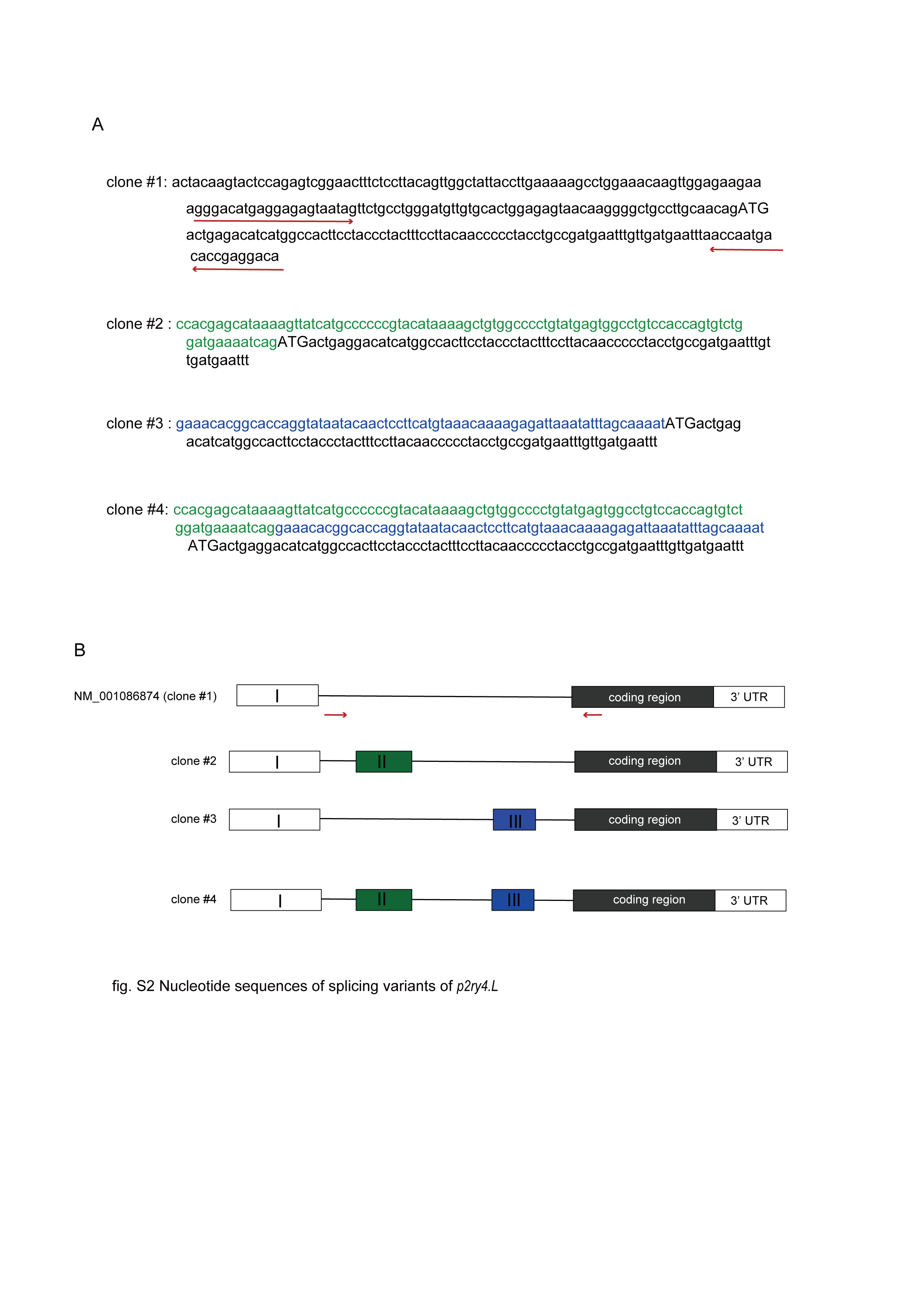

Supplement: Supplementary file 2 [file DGD-61-186-s002.tif]

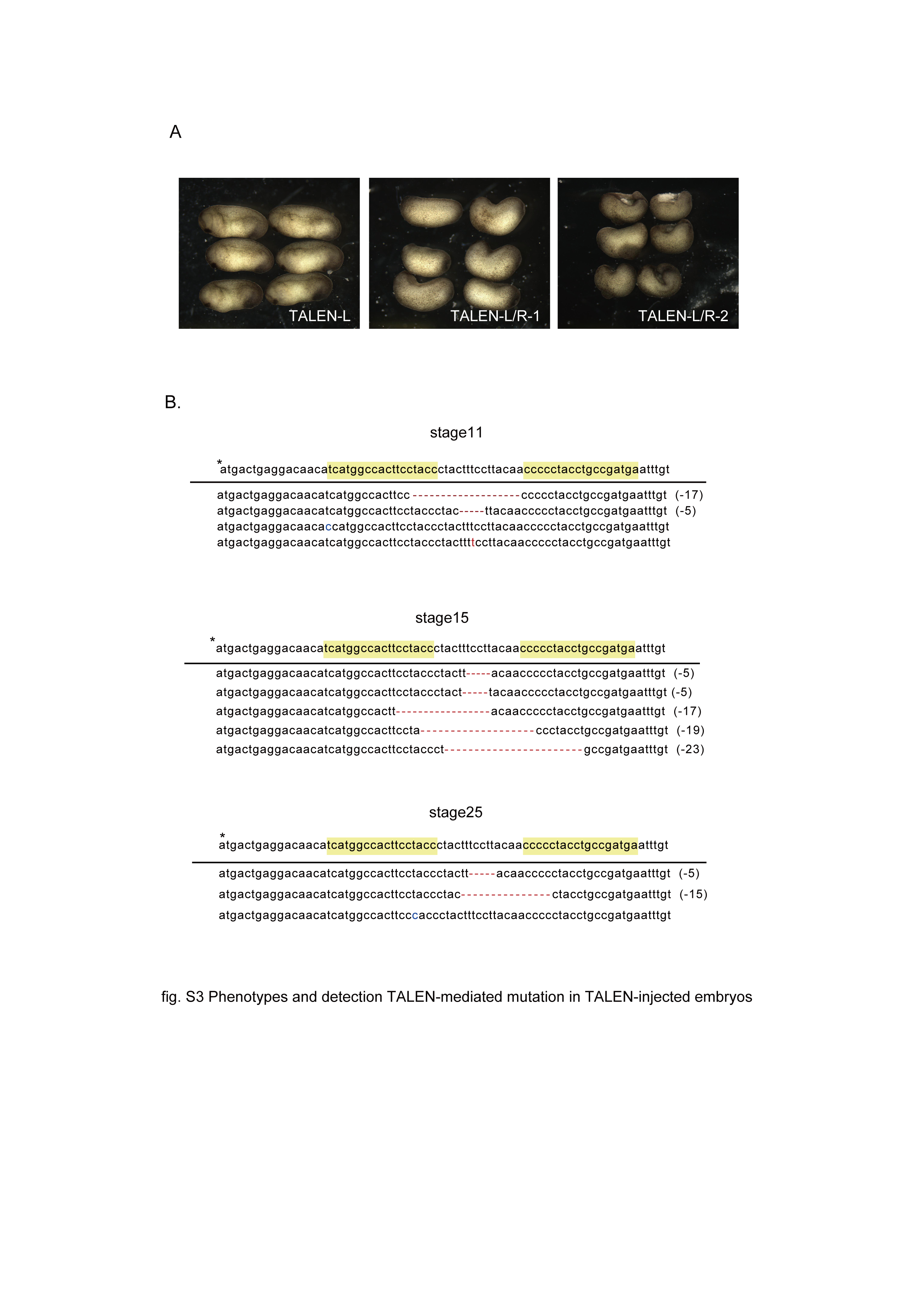

Supplement: Supplementary file 3 [file DGD-61-186-s003.tif]

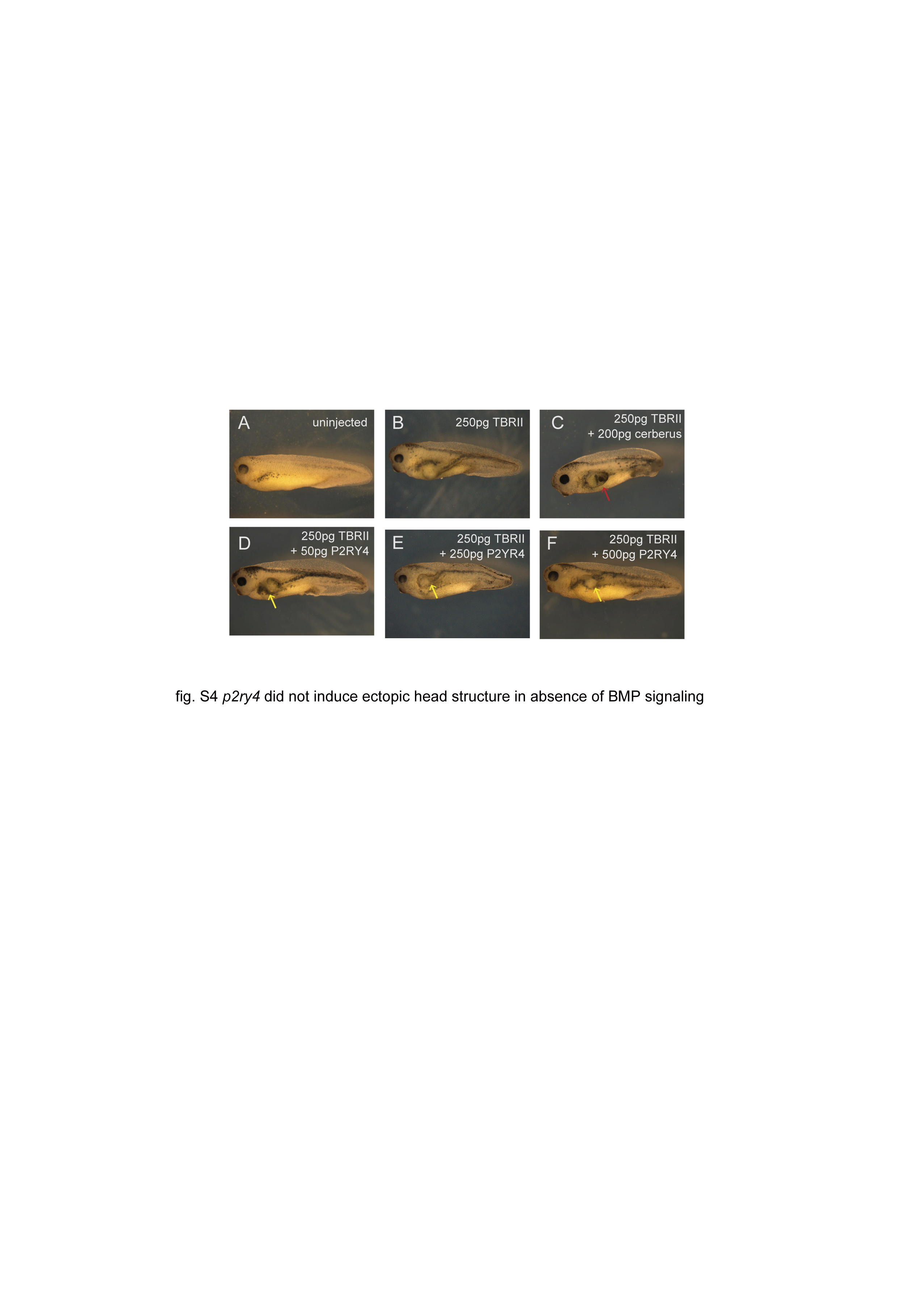

Supplement: Supplementary file 4 [file DGD-61-186-s004.tif]

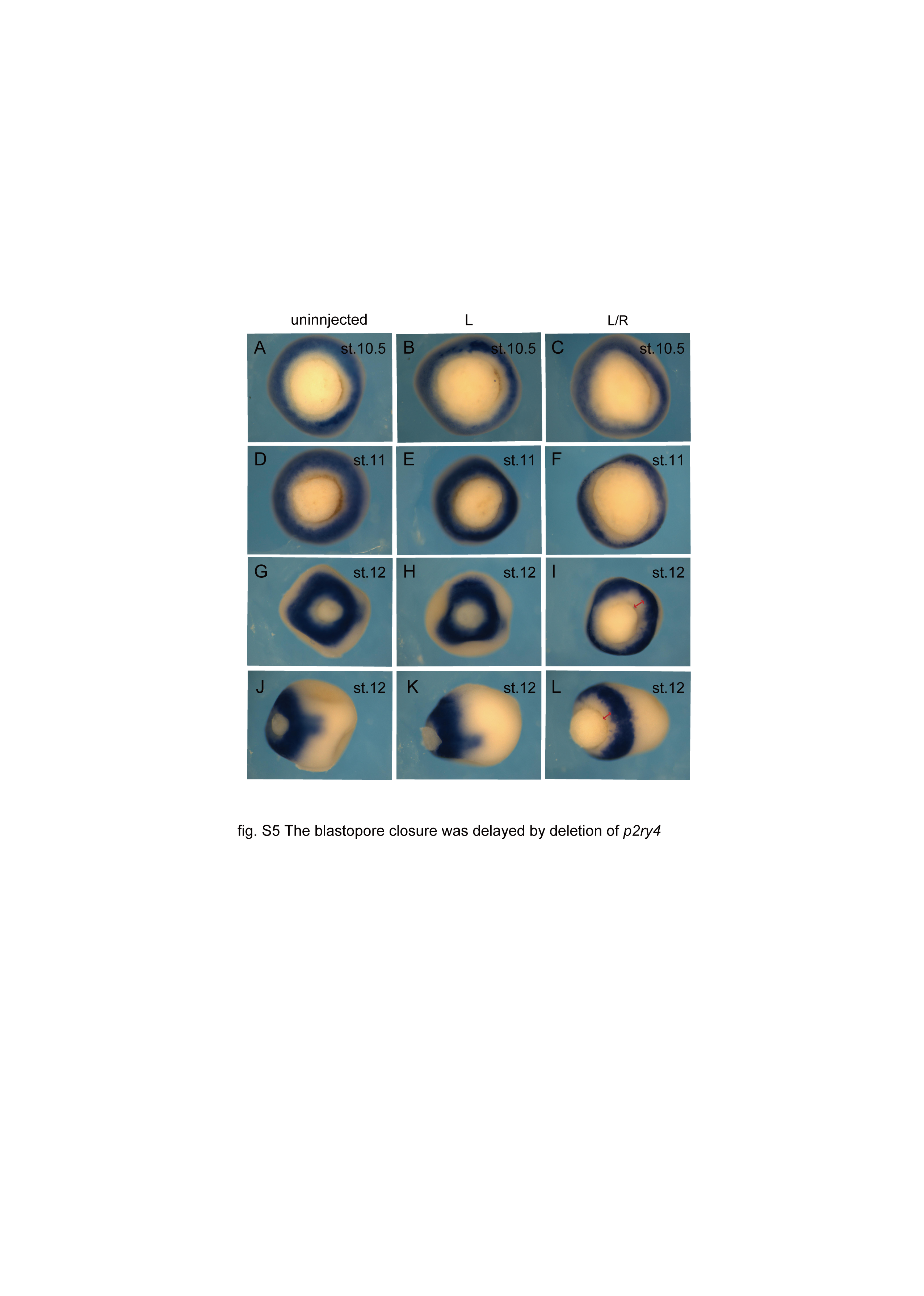

Supplement: Supplementary file 5 [file DGD-61-186-s005.tif]

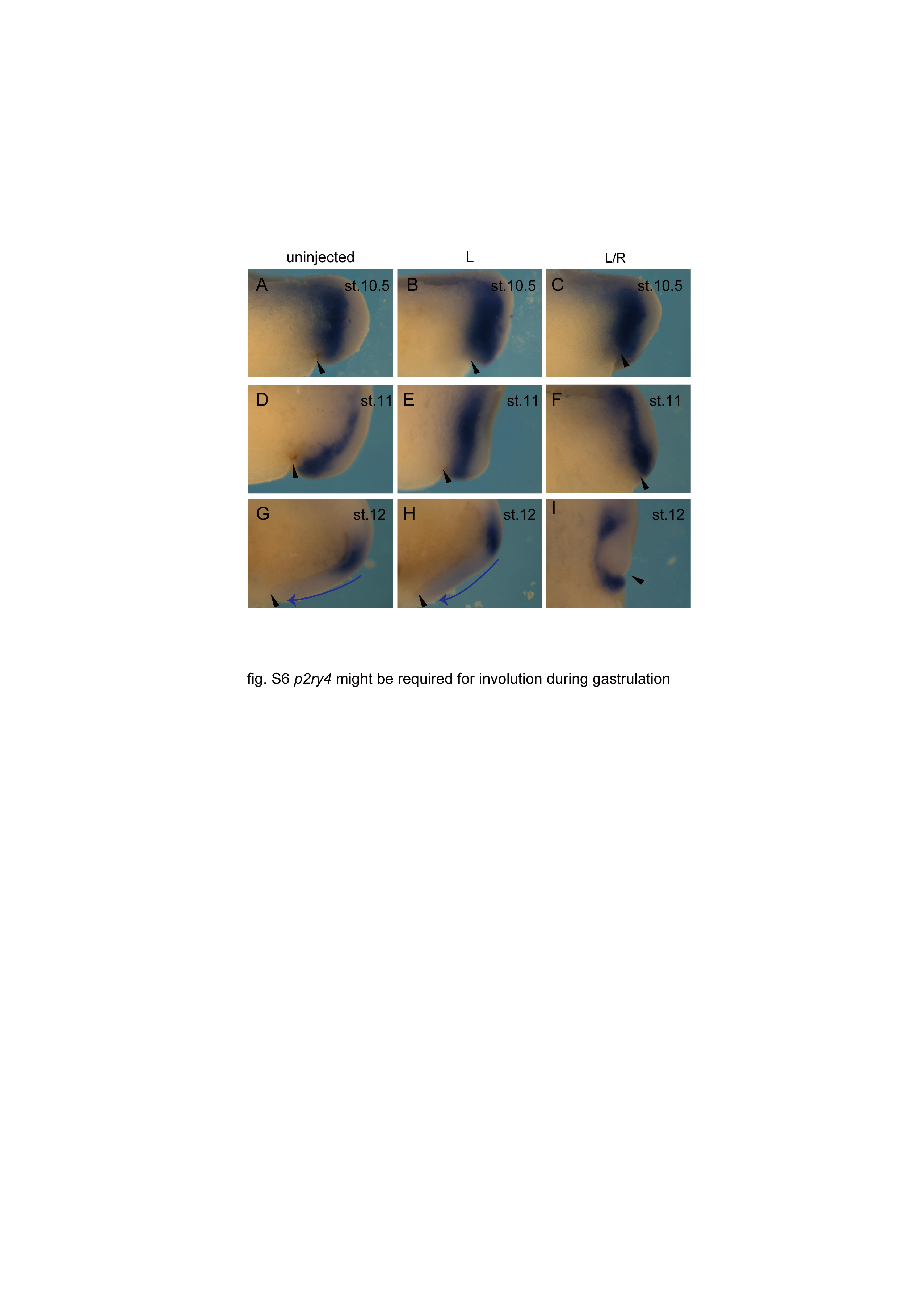

Supplement: Supplementary file 6 [file DGD-61-186-s006.tif]
